# Apomorphine Sublingual Film Compared with Subcutaneous Apomorphine for OFF Episodes in Parkinson’s Disease: An Open-Label, Randomized, Crossover Study

**DOI:** 10.3233/JPD-230072

**Published:** 2023-12-19

**Authors:** Fabrizio Stocchi, Olivier Rascol, Werner Poewe, K. Ray Chaudhuri, Jan Kassubek, Lydia Lopez Manzanares, Yi Zhang, Alyssa Bowling, Eric Pappert, Stacy Wu, for the CTH-302 Study Investigators

**Affiliations:** a University San Raffaele Roma and Institute for Research and Medical Care IRCCS San Raffaele Pisana, Rome, Italy; b University Hospital of Toulouse, University of Toulouse and INSERM, Faculté de Médecine, Toulouse, France; c Department of Neurology, Medical University Innsbruck, Innsbruck, Austria; d Parkinson’s Foundation Centre of Excellence, King’s College Hospital and King’s College London, Denmark Hill, London, UK; e Department of Neurology, University Hospital Ulm, Ulm, Germany; f La Princesa University Hospital, Madrid, Spain; g Sunovion Pharmaceuticals Inc., Marlborough, MA, USA; h Neurology Associates, San Antonio, TX, USA

**Keywords:** Apomorphine sublingual film, subcutaneous apomorphine, carbidopa/levodopa, Parkinson’s disease, OFF episode

## Abstract

**Background:**

Apomorphine sublingual film (SL-APO) and subcutaneous apomorphine (SC-APO) have been used for the treatment of OFF episodes in Parkinson’s disease (PD). No study has prospectively compared efficacy and safety of these formulations.

**Objective:**

To compare SL-APO with SC-APO for treatment of OFF episodes in PD.

**Methods:**

An open-label, randomized, crossover study assessed SL-APO versus SC-APO in patients with PD and OFF episodes (*N* = 113). Doses were optimized in randomly assigned order. SL-APO dose initiation (10 mg) occurred in clinic; further dose optimization (15–30 mg; 5-mg increments) occurred primarily at home. SC-APO dosing (2–6 mg; 1-mg increments) occurred entirely in clinic. After a 3–7-day washout, patients were randomized 1 : 1 to 4 weeks of treatment with their optimized dose of SL-APO or SC-APO, followed by washout and 4 weeks of crossover treatment.

**Results:**

Propensity score matching applied on 159 patients (STN-DBS *n* = 75, MED *n* = 84) resulted in 40 patients in each treatment group. At 36-month follow-up, STN-DBS led to significantly better PDSS and PDQ-8 change scores, which were significantly correlated. We observed no significant effects for HADS and no significant correlations between change scores in PDSS, HADS, and LEDD.

**Conclusions:**

We report Class IIb evidence of beneficial effects of STN-DBS on quality of sleep at 36-month follow-up, which were associated with QoL improvement independent of depression and dopaminergic medication. Our study highlights the importance of sleep for assessments of DBS outcomes.

**Results:**

No difference was observed between SL-APO and SC-APO for change from predose to 90 minutes postdose in Movement Disorder Society Unified Parkinson’s Disease Rating Scale Part III score at week 4 (primary endpoint), assessed by a blinded rater (–13.6 vs. –13.8, respectively; *p* = NS). Overall, 72.2% of patients preferred SL-APO compared with SC-APO/no preference (*p* = 0.0002) per the Treatment Preference Questionnaire (secondary endpoint). Patients reported greater satisfaction with SL-APO compared with SC-APO, per mean scores of convenience (73.7 vs. 53.5) and global satisfaction (63.9 vs. 57.6) on the Treatment Satisfaction Questionnaire for Medication (other endpoint). The safety profiles of both treatments were generally comparable and were well-tolerated.

**Conclusions:**

Patients reported overall preference for and greater satisfaction with SL-APO over SC-APO.

## INTRODUCTION

Oral carbidopa/levodopa (CD/LD) is the cornerstone of Parkinson’s disease (PD) treatment [1, 2]. Over time, many patients develop OFF episodes, defined as periods where motor and/or nonmotor symptoms reappear or worsen [3]. Different strategies are applied to manage OFF episodes [4–8]; however, despite CD/LD adjustments, combinations of different drugs, and/or use of “ON-extenders,” patients may continue to experience 4–5 h of daily OFF time [7–17].

Apomorphine, a D1-family and D2-family agonist with an affinity similar to dopamine, is used for the treatment of OFF episodes in patients with PD [2, 18, 19]. Apomorphine has been shown to provide motor benefit that is comparable to CD/LD, but with a more rapid onset of effect [2]. Currently, the following two apomorphine formulations are used for the acute, intermittent treatment of OFF episodes: subcutaneous apomorphine (SC-APO), the “classic” formulation, and apomorphine sublingual film (SL-APO), a novel formulation [6]. In the pivotal SL-APO study, a significant improvement in Movement Disorder Society Unified Parkinson’s Disease Rating Scale (MDS-UPDRS) Part III score from predose to 30 min postdose was observed with SL-APO versus placebo (–11.1 vs. –3.5; *p* < 0.0002) [20]. In the pivotal study for SC-APO, the Unified Parkinson’s Disease Rating Scale (UPDRS) Part III score was significantly improved from baseline for SC-APO versus placebo at 20 min postdose (–23.9 vs. –0.1; *p* < 0.001) [21]. Both SL-APO and SC-APO were associated with dopaminergic class-related adverse events (AE), as well as route of administration AEs [20, 21].

SC-APO is a reliable, effective treatment option for managing OFF episodes; however, its utilization has been limited. SC-APO is administered by subcutaneous injection and involves a multistep product assembly for administration, which may be challenging for patients experiencing decreased motor function [20]. SL-APO was designed to maintain the clinical benefits associated with SC-APO while addressing limitations by providing a convenient sublingual formulation with a rapid treatment effect that circumvents the challenges associated with an injection [20]. A previous study showed the extent of exposure based on area under the concentration-time curve (AUC) was found to be comparable for both SL-APO and SC-APO [22]. However, no study has prospectively compared the efficacy, safety, and patient preference of SL-APO and SC-APO for the treatment of OFF episodes.

## METHODS

### Study design and participants

This multicenter, open-label, randomized, crossover study assessed SL-APO compared with SC-APO in patients with PD and OFF episodes and was conducted in Europe (EudraCT: 2016-003456-7). Herein we provide a brief review of methods, with full methodology available in the Supplementary Methods. Eligible patients were ≥18 years of age with idiopathic PD responsive to and being treated with stable doses of CD/LD and any additional PD medications for ≥4 weeks (>8 weeks for monoamine oxidase-B inhibitors), were stage 1–3 by modified Hoehn and Yahr scale when ON, had ≥1 OFF episode/day and ≥2 h of total daily OFF time, and had a Mini-Mental State Examination score >25. Key exclusion criteria included atypical or secondary parkinsonism; major psychiatric disorder; mouth cankers/sores; prior device-aided treatments; permanent discontinuation of prior SC-APO administration or prior exposure to SL-APO; currently taking selective 5-HT_3_ antagonists, selective dopamine antagonists (excluding quetiapine or clozapine), or dopamine-depleting agents; and history of clinically significant impulse control disorders, symptomatic orthostatic hypotension requiring medication, or severe dyskinesia based on MDS-UPDRS Part IV.

The study was designed, conducted, and monitored in accordance with the World Medical Association Declaration of Helsinki (1989) and International Council for Harmonisation guidelines. The study protocol and study procedures were approved by institutional review boards and independent ethics committees at each study site (Supplementary Methods).

### Procedures

**Fig. 1:**
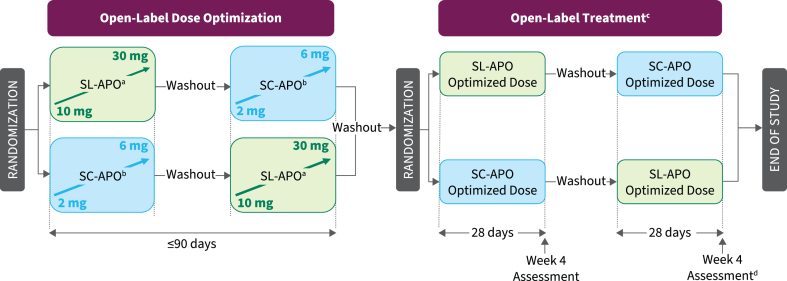
Study design. ^a^Patients in a practically defined OFF received 10 mg of SL-APO in the clinic and if a FULL ON (defined as the period when medication provided benefit with regard to mobility, stiffness, and slowness and the patient having adequate motor function to perform normal daily activities) was not achieved within 30 min, up-titration (5-mg dose increases; 30-mg dose maximum) during subsequent practically defined OFF episodes could continue at home without in-person observation. If a FULL ON was achieved at home, patients returned to the clinic for a dose-confirmation visit, during which the investigator could adjust the dose, if necessary. ^b^Patients in a practically defined OFF received 2 mg of SC-APO in the clinic and if a FULL ON was not achieved within 30 min, up-titration (1-mg dose increases; 6-mg dose maximum) during subsequent OFF episodes continued in the clinic. ^c^Patients could administer study drug for up to 5 OFF episodes per day, with doses separated by ≥2 h. ^d^Treatment Preference Questionnaire was performed at week 4 after both regimens had been completed. SC-APO, subcutaneous apomorphine; SL-APO, apomorphine sublingual film.

The study consisted of open-label dose-optimization and open-label treatment phases (Fig. 1). During dose optimization, doses of both medications were optimized in a randomly assigned order to determine the dose that provided a FULL ON (benefit with regard to mobility, stiffness, and slowness and the patient having adequate motor function to perform normal daily activities) within 30 min when patients were in a practically defined OFF (no antiparkinson medications after midnight the night before). A washout of 3–7 days occurred between treatment periods.

SL-APO (Supplementary Figure 1) was initiated at 10 mg in clinic, with monitoring of vital signs assessed predose and within 60 min postdose. If a FULL ON was not achieved within 30 min, up-titration (5-mg dose increases; 30-mg dose maximum) during subsequent practically defined OFF episodes could continue at home without direct in-person observation or vital sign monitoring. Clinic staff contacted patients daily by phone during home dose optimization to monitor progress and assess tolerability based on patient self-report. An in-clinic dose-confirmation visit took place after the patient identified their optimal dose at home to confirm the effectiveness and tolerability of the selected dose. If the investigator determined the FULL ON response to be inadequate (based on effectiveness) or there were tolerability concerns, dose adjustment could continue either in clinic or at home, followed by additional dose-confirmation visits, as needed.

Dose optimization of SC-APO (Supplementary Figure 2) was initiated at 2 mg for patients with no previous SC-APO experience and took place entirely in clinic under direct supervision, with monitoring of vital signs predose and within 60 min postdose. Patients with previous SC-APO experience completed a washout of ≥1 day before study enrollment and began dose optimization at the same dose of SC-APO they were taking before screening. If a FULL ON was not achieved within 30 min, up-titration in 1-mg increments continued in clinic during subsequent OFF episodes, no earlier than 60 min after the prior dose. If a FULL ON was not achieved at the 4-mg dose, the patient returned to the clinic the next day. Dose optimization continued in the same manner until a FULL ON was achieved (maximum 6 mg).

Initially, use of the antiemetic domperidone was optional if clinically warranted and was not to be used prophylactically. After a protocol amendment, domperidone use remained optional but could also be used prophylactically or if clinically warranted at the discretion of the investigator. If initiated, antiemetic therapy was discontinued when judged clinically appropriate.

After a 3- to 7-day washout, patients entered the treatment phase and were randomized in a 1 : 1 ratio to 4 weeks of treatment with the optimized dose of SL-APO or SC-APO, followed by a washout and 4 weeks of crossover treatment. Patients continued their regular PD medication regimen and could self-administer study treatment for ≤5 OFF episodes per day when needed. Clinic visits occurred every 2 weeks, with the patient presenting in a practically defined OFF episode.

### Evaluations

The primary efficacy endpoint was change from predose to 90 min postdose in MDS-UPDRS Part III score after 4 weeks of dosing in each crossover period, assessed in clinic by a rater blinded to treatment assignment. The blind was maintained by ensuring that the rater did not witness in-clinic dosing, that visible injection sites were covered, and that source data and electronic clinical report forms were protected. Because SL-APO can leave a blue residue on the tongue, a sublingual placebo was administered upon SC-APO in-clinic dosing. Secondary endpoints were evaluated in a hierarchical order and included the following: investigator-rated durability of response (defined as investigator-confirmed achievement of a FULL ON within 30 min postdose and maintenance of that response at 90 min postdose); treatment preference for SL-APO, measured with a 9-item patient self-reported Treatment Preference Questionnaire (TPQ [23]; Supplementary Table 1); patient-rated durability of response; and Patient Global Impression of Change (PGI-C). Other endpoints included change in MDS-UPDRS Part III score over time (15–120 min); investigator-rated time to FULL ON and time to partial ON (period of time where medication is providing some improvement with regard to mobility, stiffness, and slowness but the patient does not have adequate motor function to perform normal daily activities); and patients’ general level of satisfaction with medication using the validated 14-item Treatment Satisfaction Questionnaire for Medication (TSQM) [24].

**Fig. 2:**
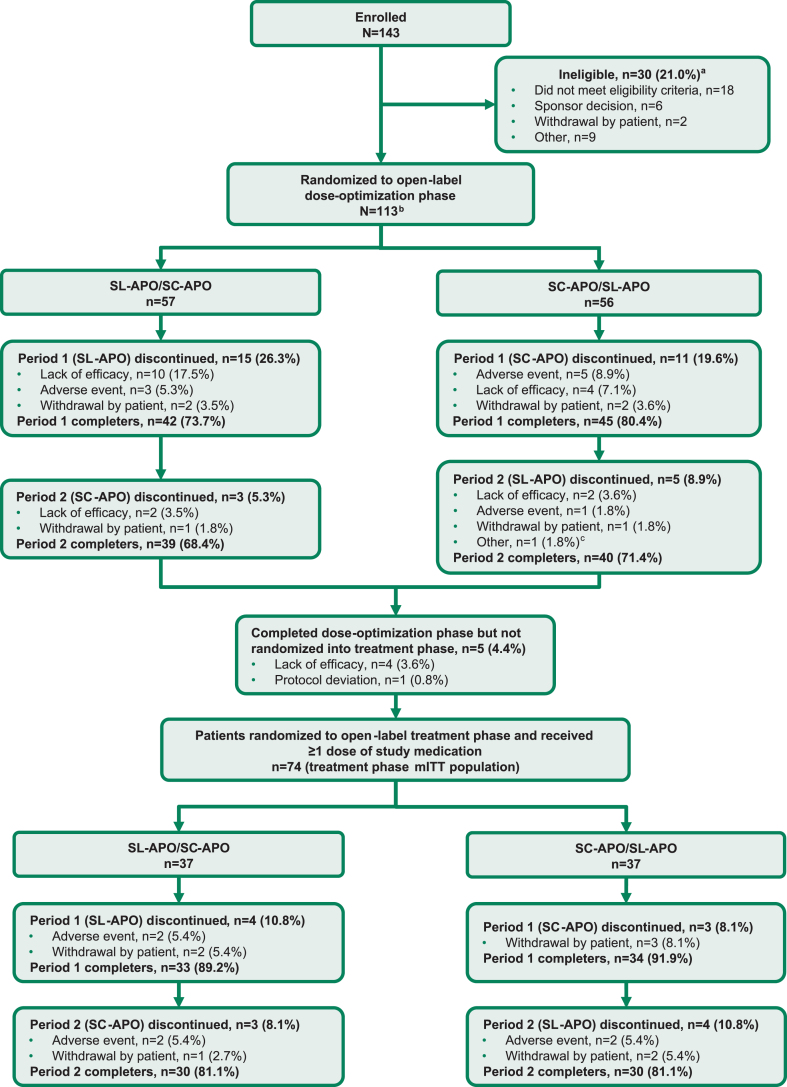
Consort flow diagram. ^a^Patients can be included in more than 1 category of ineligibility. ^b^The dose-optimization safety population was defined as all patients who received ≥1 dose of study medication in the open-label dose-optimization phase (*N* = 112). ^c^Early termination at sponsor request. mITT, modified intention-to-treat; SC-APO, subcutaneous apomorphine; SL-APO, apomorphine sublingual film.

Pharmacokinetic (PK) concentration-time data for apomorphine and metabolites (apomorphine sulfate, norapomorphine) were evaluated and PK parameters, including maximum observed plasma concentration (C_max_), time to maximum plasma concentration (t_max_), AUC, and parent-to-metabolite ratios of C_max_ and AUC were estimated by noncompartmental methods from plasma samples using actual elapsed time from dosing. PK sample collection took place just before dosing and at 15, 30, 60, 90, 120, 180, and 240 min postdose (±5 min) at clinic visits during the treatment phase.

Unblinded safety evaluations conducted during both study phases included assessments of AEs, physical examinations, 12-lead electrocardiograms, and vital signs.

### Statistical analysis

The primary objective of the study was to demonstrate superiority of SL-APO over SC-APO in improving motor function assessed as change from predose to 90 min postdose in MDS-UPDRS Part III score for SL-APO compared with SC-APO after 4 weeks of dosing in each crossover period (primary endpoint). The sample size calculation was based on randomization of 106 patients in the dose-optimization phase and ≥80 patients in the treatment phase, with ≥55 patients expected to complete treatment; this would provide 90% power to detect a mean treatment difference between SL-APO and SC-APO of 5.5 points for the change in MDS-UPDRS Part III score, assuming a standard deviation (SD) of 12 points for the period differences in treatment. The primary endpoint was analyzed in the treatment phase modified intention-to-treat (mITT) population (all patients who were randomized and received ≥1 dose of either study drug in the treatment phase) and was compared between treatment groups using a linear mixed model, with treatment group, visit week (0, 2, 4), treatment by visit week interaction, treatment phase sequence, and period as fixed factors and the week 0 visit predose MDS-UPDRS Part III score as a covariate. The primary and secondary endpoints were tested in a hierarchical order to maintain an overall type I error rate of 0.05. AEs were summarized descriptively for both the dose-optimization and treatment phase safety populations (all patients who received ≥1 dose of either study drug for each phase).

## RESULTS

### Patients

Patients were enrolled and evaluated from December 2018 through August 2021. A total of 143 patients were assessed for eligibility. Of these, 113 patients were randomized into the open-label dose-optimization phase (57 patients randomized to receive SL-APO followed by SC-APO; 56 patients randomized to receive SC-APO followed by SL-APO; Fig. 2). A total of 112 patients received ≥1 dose of either study medication during dose optimization (dose-optimization phase safety population). A similar proportion of patients completed both periods of the dose-optimization phase for each treatment sequence (SL-APO/SC-APO, 68.4%; SC-APO/SL-APO, 71.4%). Five patients completed dose optimization but were not randomized into the open-label treatment phase (4 due to lack of efficacy, 1 due to protocol deviation). A total of 74 patients were randomized into the treatment phase (treatment phase mITT population: 37 patients randomized to receive SL-APO followed by SC-APO; 37 patients randomized to receive SC-APO followed by SL-APO). The same proportion of patients completed both periods of the treatment phase for each treatment sequence (SL-APO/SC-APO, 81.1%; SC-APO/SL-APO, 81.1%).

At study baseline (*N* = 112), patient mean age was 64.4 years, and 69.6% were male. Patients were diagnosed with PD a mean of 9.2 years prior, experienced a mean of 4.1 OFF episodes per day, and were prescribed a mean daily levodopa dose of 737.6 mg (Table 1). Thirteen (11.6%) patients had received SC-APO before study enrollment, with a median dose of 4 mg (range, 2–6 mg; data not available for 1 patient) and continued on the same dose of SC-APO during the dose-optimization phase. The mean optimized dose of SL-APO for these patients was 25 mg (range, 10–30 mg).

**Table 1:** Demographic and baseline clinical characteristics^a^ (dose-optimization phase safety population)

Parameter	Overall (*N* = 112)
Age, y, mean (SD)	64.4 (8.8)
≥65 y, *n* (%)	60 (53.6)
Male, *n* (%)	78 (69.6)
Modified Hoehn and Yahr score when ON, *n* (%)
1 or 1.5	17 (15.2)
2 or 2.5	76 (67.8)
3	19 (17.0)
MDS-UPDRS Part III score when OFF at screening, mean (SD)	51.1 (13.1)
Total daily levodopa dose, mg, mean (SD)	737.6 (431.9)
Time since PD diagnosis, y, mean (SD)	9.2 (4.2)
Time since motor fluctuations started, y, mean (SD)	3.5 (2.5)
Number of OFF episodes experienced per day, mean (SD)	4.1 (1.3)
Concomitant PD medications, *n* (%)
Dopamine agonists	91 (81.3)
Monoamine oxidase-B inhibitors	59 (52.7)
Catechol-O-methyltransferase inhibitors	36 (32.1)
Amantadine	21 (18.8)

^a^Determined by detailed assessment of patient history. MDS-UPDRS, Movement Disorder Society Unified Parkinson’s Disease Rating Scale; PD, Parkinson’s disease; SD, standard deviation.

At randomization into the treatment phase, 32.4% (23/71) of patients received the highest dose of SL-APO (30 mg) and 21.4% (15/70) of patients received the two highest doses of SC-APO (5 or 6 mg; Table 2). The mean (SD) number of daily doses during the treatment phase based on patient diaries was 1.7 (1.0) for SL-APO and 1.5 (1.0) for SC-APO.

**Table 2:** Extent of exposure

		SL-APO (*n* = 102)				
*n* (%)	10 mg	15 mg	20 mg	25 mg	30 mg
Dose at randomization for treatment phase (*n* = 71)^a^	11 (15.5)	7 (9.9)	15 (21.1)	15 (21.1)	23 (32.4)
		SC-APO (n=97)				
*n* (%)	2 mg	3 mg	4 mg	5 mg	6 mg
Dose at randomization for treatment phase (*n* = 70)^a^	21 (30.0)	20 (28.6)	14 (20.0)	8 (11.4)	7 (10.0)

^a^Percentages are based on the number of patients randomized into the open-label treatment phase and received a dose of treatment. SC-APO, subcutaneous apomorphine; SL-APO, apomorphine sublingual film.

### Efficacy

**Fig. 3:**
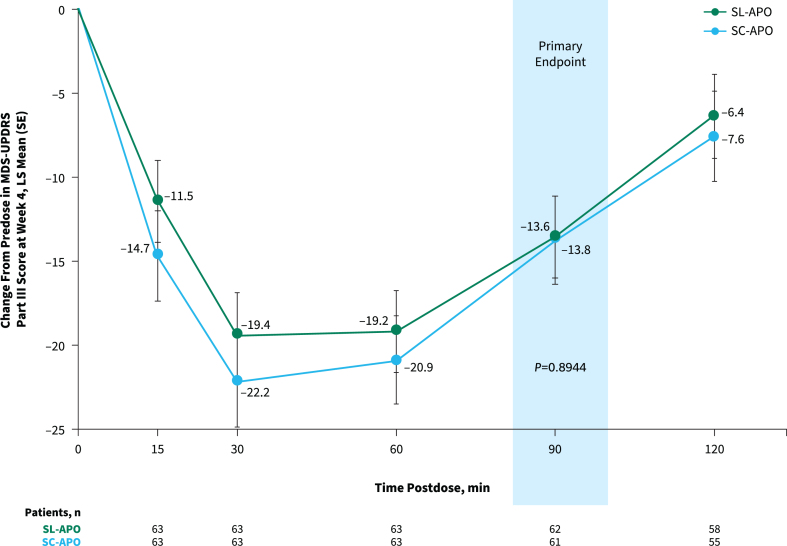
Change from predose in MDS-UPDRS Part III score at week 4 over time (treatment phase mITT population). Data represent MDS-UPDRS Part III scores achieved with the optimized dose of each apomorphine formulation at week 4 of the treatment phase. LS, least squares; MDS-UPDRS, Movement Disorder Society Unified Parkinson’s Disease Rating Scale; mITT, modified intention-to-treat; SC-APO, subcutaneous apomorphine; SE, standard error; SL-APO, apomorphine sublingual film.

The primary endpoint of change from predose to 90 min postdose in MDS-UPDRS Part III score at week 4 was comparable for SL-APO and SC-APO (least squares [LS] mean [standard error], –13.6 [1.5] vs. –13.8 [1.5]; *p* = 0.8944; Fig. 3). As the primary endpoint was not significant and efficacy analyses were performed in a hierarchical order, nominal *p* values are provided for subsequent endpoints. Motor responses at all time points assessed through 120 min were similar, and the LS mean treatment differences at week 4 for all other time points did not reach significance, except at 15 min (–3.2; *p* = 0.025) and 30 min (–2.8; *p* = 0.039) postdose (both in favor of SC-APO). Investigator-rated time to FULL ON and time to partial ON for SL-APO and SC-APO were similar across all time points at week 4 (Supplementary Figure 3). Investigator-rated FULL ON at week 4 was achieved by 85.7% of patients treated with SL-APO and 92.2% of patients treated with SC-APO, with a median time to FULL ON (95% CI) of 30.0 min (27.0–31.0) and 30.0 min (24.0–31.0), respectively. Median (95% CI) time to partial ON at week 4 was 15.0 min (15.0–18.0) for SL-APO and 15.0 min (not evaluable [NE]–NE) for SC-APO. A numerically greater proportion of patients treated with SC-APO versus SL-APO achieved investigator-rated FULL ON and partial ON at 15 min postdose at week 4 (FULL ON: 15.6% vs. 4.8%, respectively; partial ON: 62.5% vs. 51.6%; Supplementary Figure 3).

**Fig. 4:**
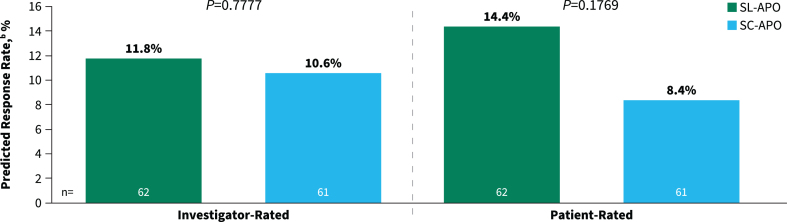
Investigator- and patient-rated durability of response^a^ at week 4 (treatment phase mITT population). ^a^Defined as patient achievement of a FULL ON within 30 min postdose and maintenance of that response at 90 min postdose. mITT, modified intention-to-treat; SC-APO, subcutaneous apomorphine; SL-APO, apomorphine sublingual film.

The secondary endpoints of investigator- and patient-rated durability of response were comparable between SL-APO and SC-APO at week 4 (investigator-rated predicted response rate, 11.8% vs. 10.6% [*p* = 0.7777]; patient-rated predicted response rate, 14.4% vs. 8.4% [*p* = 0.1769]; Fig. 4). Additionally, similar proportions of patients reported improvement on the PGI-C with SL-APO compared with SC-APO (83.1% vs. 77.1%; *p* = 0.3922).

The secondary endpoint of patient preference for SL-APO over SC-APO was evaluated using the TPQ at study end, after patients completed both treatment periods. When patients were presented with the following statement: “Overall, the treatment I prefer for my OFF episodes is,” 72.2% preferred SL-APO compared with SC-APO/no preference (95% CI, 61.9–82.6; *p* < 0.0002). Patients reported greater satisfaction for SL-APO compared with SC-APO on the TSQM (exploratory endpoint) based on mean (SD) scores for convenience (73.7 [17.0] vs. 53.5 [19.1]) and global satisfaction (63.9 [24.9] vs. 57.6 [22.0]); comparable scores were observed for effectiveness (61.0 [21.7] vs. 61.4 [20.1]) and side effects (76.7 [27.9] vs. 75.4 [27.2]).

### PK

The t_max_ of apomorphine occurred later for SL-APO compared with SC-APO (0.5–1.0 h vs. 0.3–0.4 h, respectively). The t_max_ of associated metabolites also occurred later with SL-APO compared with SC-APO (1.0–2.1 h vs. 0.5–2.0 h, respectively). Apomorphine C_max_ and AUC were similar between comparable doses of SL-APO and SC-APO. Increases in C_max_ and AUC were dose proportional with SC-APO and approximately dose proportional with SL-APO. The apomorphine relative bioavailability of SL-APO to SC-APO was approximately 14% based on C_max_/dose and approximately 20% based on AUC/dose. Variability in PK parameters after both SL-APO and SC-APO administration ranged from moderate (≥30% coefficient of variation [CV]) to <60% CV) to high (≥60% CV).

### Safety

The rates of overall AEs were generally comparable between SL-APO and SC-APO (dose-optimization phase: 62.7% vs. 55.7%, respectively; treatment phase: 53.5% vs. 68.6%; Table 3). In the subgroup of patients with prior SC-APO exposure (*n* = 13), rates of AEs with SL-APO and SC-APO were 46.2% and 58.3%, respectively, during dose optimization and 30.0% and 55.6%, respectively, during the treatment phase.

**Table 3:** Overall summary of AEs (dose-optimization and treatment phase safety populations)

	Open-label dose-optimization phase		Open-label treatment phase	
	SL-APO	SC-APO	SL-APO	SC-APO
*n* (%)	(*n* = 102)	(*n* = 97)	(*n* = 71)	(*n* = 70)
Any AEs	64 (62.7)	54 (55.7)	38 (53.5)	48 (68.6)
AEs leading to discontinuation	4 (3.9)	5 (5.2)	4 (5.6)	2 (2.9)
SAEs	0	1 (1.0)	1 (1.4)	1 (1.4)
AEs leading to death	0	0	0	0

AE, adverse event; SAE, serious adverse event; SC-APO, subcutaneous apomorphine; SL-APO, apomorphine sublingual film.

The most common AEs during dose optimization for SL-APO versus SC-APO were nausea (31.4% vs. 22.7%), somnolence (8.8% vs. 13.4%), and fatigue (5.9% vs. 10.3%; Table 4). During dose optimization, the majority of AEs of nausea were mild to moderate for both formulations, and similar rates of severe AEs of nausea were observed with SL-APO (2.0%) compared with SC-APO (1.0%). Nausea resulted in drug withdrawal in similar proportions of patients receiving SL-APO (2.0%) and SC-APO (1.0%) during dose optimization. No serious AEs of nausea were observed for either SL-APO or SC-APO during dose optimization. Rates of vomiting during dose optimization were relatively low for both SL-APO (4.9%) and SC-APO (4.1%). In the treatment phase, the most common AEs for SL-APO versus SC-APO were nausea (14.1% vs. 15.7%), dyskinesia (11.3% vs. 20.0%), and injection site hematoma (0 vs. 27.1%; Table 4).

**Table 4:** Most common (≥5%) adverse events in any treatment group (dose-optimization and treatment phase safety populations)

	Open-label		Open-label	
	dose-optimization phase		treatment phase	
	SL-APO	SC-APO	SL-APO	SC-APO
*n* (%)	(*n* = 102)	(*n* = 97)	(*n* = 71)	(*n* = 70)
Nausea	32 (31.4)^a^	22 (22.7)^a^	10 (14.1)	11 (15.7)
Dizziness	10 (9.8)	4 (4.1)	2 (2.8)	3 (4.3)
Somnolence	9 (8.8)	13 (13.4)	3 (4.2)	4 (5.7)
Dyskinesia	8 (7.8)	7 (7.2)	8 (11.3)	14 (20.0)
Fatigue	6 (5.9)	10 (10.3)	4 (5.6)	4 (5.7)
Vomiting	5 (4.9)	4 (4.1)	1 (1.4)	2 (2.9)
Orthostatic hypotension	4 (3.9)	5 (5.2)	3 (4.2)	4 (5.7)
Yawning	3 (2.9)	5 (5.2)	0	1 (1.4)
Fall	1 (1.0)	1 (1.0)	4 (5.6)	1 (1.4)
Injection site erythema	0	6 (6.2)	1 (1.4)	5 (7.1)
Injection site hematoma	0	2 (2.1)	0	19 (27.1)

^a^Antiemetic therapy could be initiated if clinically warranted. During the dose-optimization phase, 56.9% of patients on SL-APO and 53.6% on SC-APO did not use antiemetics, with nausea being reported during dose optimization by 15.5% and 9.6% of these patients, respectively. During the treatment phase, 56.3% of patients on SL-APO and 57.1% on SC-APO did not use antiemetics, with nausea being reported during the treatment phase by 5.0% and 5.0% of these patients, respectively. SC-APO, subcutaneous apomorphine; SL-APO, apomorphine sublingual film.

Overall, there was no apparent dose-dependent relationship for AEs with SL-APO or SC-APO formulation in either study phase. In both the dose optimization phase and treatment phases, the frequency of AEs leading to discontinuation were generally comparable between the two formulations (Table 5). Serious AEs occurred in a low number of patients and no deaths were reported during the study.

**Table 5:** Patients in any treatment group with adverse events leading to discontinuation (dose-optimization and treatment phase safety populations)

	Open-label		Open-label	
	dose-optimization phase		treatment phase	
	SL-APO	SC-APO	SL-APO	SC-APO
*n* (%)	(*n* = 102)	(*n* = 97)	(*n* = 71)	(*n* = 70)
Chills	0	0	1 (1.4)	0
Dysphagia	0	0	1 (1.4)	0
Fatigue	0	1 (1.0)	0	0
Freezing phenomenon	0	0	1 (1.4)	0
Glossodynia	0	0	1 (1.4)	0
Hyperhidrosis	1 (1.0)	0	0	0
Hypotension	1 (1.0)	1 (1.0)	0	0
Lip swelling	0	0	1 (1.4)	0
Nausea	2 (2.0)	1 (1.0)	0	0
ON and OFF phenomenon	0	0	1 (1.4)	0
Orthostatic hypotension	0	1 (1.0)	0	1 (1.4)
Somnolence	0	0	0	1 (1.4)
Swollen tongue	0	0	1 (1.4)	0
Syncope	0	1 (1.0)	0	0
Vomiting	2 (2.0)	2 (2.1)	0	0

SC-APO, subcutaneous apomorphine; SL-APO, apomorphine sublingual film.

## DISCUSSION

This open-label, randomized, crossover study was designed to meet regulatory requirements as a registrational study in the European Union. The purpose was to compare efficacy, safety, tolerability, and patient preference for SL-APO and SC-APO, with the 90-min primary endpoint chosen based on prior PK studies [22]. While the study did not achieve its primary endpoint or confirm the hypothesis of superiority of SL-APO over SC-APO for MDS-UPDRS Part III scores at 90 min postdose (defined as an improvement of 3.25 points [25]), valuable lessons can be gathered from the results. Overall, SL-APO and SC-APO demonstrated comparable efficacy across multiple endpoints. Motor responses on the MDS-UPDRS Part III at all time points assessed through 120 min were generally comparable between the two formulations. However, at 15 and 30 min postdose, the differences in MDS-UPDRS Part III scores were nominally significant and favored SC-APO, which may be expected based on the PK profile of SC-APO compared with SL-APO. More than 85% of patients in both groups achieved FULL ON by 90 min postdose, demonstrating that both formulations are efficacious. Investigator- and patient-rated durability of response and patient-reported improvement on the PGI-C were comparable for SL-APO and SC-APO. Patients reported an overall preference for and greater global satisfaction and convenience with SL-APO compared with SC-APO.

Prior studies have demonstrated a rapid onset of effect of SL-APO as early as 15 min postdose, which was chosen as the earliest time point against which differences between SL-APO and SC-APO were measured in this study [20, 26, 27]. Although the median time to partial ON and FULL ON were comparable for SC-APO and SL-APO at 15 and 30 min, results on other endpoints suggested a faster onset of action for SC-APO. A nominally significant difference in motor response favoring SC-APO was seen at 15 and 30 min postdose; at 15 min postdose, the difference nearly met the clinically important difference of improvement in the MDS-UPDRS Part III score [25]. Further, there were numerically greater proportions of patients treated with SC-APO versus SL-APO who achieved FULL ON and partial ON at time points earlier than 20 min postdose. These findings were not unexpected given that SC-APO has been shown to have a faster time to peak plasma concentration compared with SL-APO, which was corroborated with current data and suggests a potentially faster time to ON for SC-APO [22]. Data in this study were not collected before 15 min postdose; thus, additional studies would be needed to demonstrate differential effects at time points before 15 min postdose. Overall, the differences in efficacy observed at time points before and at 30 min in the current study were small and did not meet the threshold of being clinically meaningful, suggesting relatively similar clinical benefit for patients with either treatment at these time points.

The 90-min time point chosen for the primary endpoint was based on differences in PK observed between SL-APO and SC-APO and previous findings from SC-APO studies. In a randomized, open-label, 3-way crossover study, C_max_ was reached approximately 0.5–1.0 h postdose for SL-APO and 0.25–0.5 h postdose for SC-APO, yet the extent of exposure based on AUC was found to be comparable for both treatments [22]. Further, a randomized, placebo-controlled study of SC-APO demonstrated no difference between SC-APO and placebo at 90 min for change from predose in UPDRS Part III score [28]. Together, these findings suggested that SL-APO may have a longer duration of effect than SC-APO. The current head-to-head study demonstrated comparable changes in MDS-UPDRS Part III scores at 60, 90, and 120 min postdose for SL-APO and SC-APO. Despite higher plasma apomorphine concentrations for SL-APO compared with SC-APO at these later time points, the clinical efficacy results do not support the hypothesis of a longer duration of effect for SL-APO and suggest a potential dissociation between the PK and pharmacodynamics of the two treatments. Therefore, rather than the actual plasma concentration, clinical efficacy may depend on whether the plasma concentration has risen above a minimum level where benefit from apomorphine would be expected.

Clinical guidance extrapolated from the results of this study suggest that while dose optimization is highly individualized, generally a factor of 5 to 10 can be used to determine a comparable dose when converting a patient from SC-APO to SL-APO. Based on prior PK studies [22], at the 2-mg dose of SC-APO, the approximate equivalent dose of SL-APO was expected to be 10 or 15 mg, while at doses of 3 and 4 mg SC-APO, the comparable dose of SL-APO was expected to be 20 and 25 mg, respectively. Five- and 6-mg doses of SC-APO were generally comparable to 30-mg doses of SL-APO. In the current study, dose optimization was based on the achievement of a FULL ON within 30 min; considering the rapid rate of absorption and earlier t_max_ for SC-APO versus SL-APO, patients treated with SC-APO at lower dose levels may have more easily reached FULL ON within 30 min of drug administration, whereas higher dose levels of SL-APO may have been needed to achieve the same effect within the same time period. Indeed, 50% of patients were optimized at the two highest doses of SL-APO (25 and 30 mg), while >50% of patients were optimized at the two lowest doses of SC-APO (2 and 3 mg).

An overall preference for SL-APO was observed based on the TPQ. These results are particularly robust considering the crossover design of the study, in which patients were treated with both formulations and provided their response regarding preference for treatment at study end. This finding was supported by a numerically higher level of satisfaction with SL-APO compared with SC-APO on the TSQM for convenience and global satisfaction. Although the TPQ was developed for this study and has not been formally validated, alignment between the TPQ and TSQM findings supports the content validity of the TPQ. These results align with a previous finding that, for OFF treatment modes associated with AEs, patients with PD reported a preference for a hypothetical sublingual film associated with mouth/lip sores versus a hypothetical injectable medication associated with injection site reactions [29]. Further, an indirect treatment comparison based on a systematic literature review suggested that SL-APO and SC-APO are comparable in efficacy but additional factors, including mode of administration, may influence treatment decisions [30]. Although a difference in dose-optimization protocols may have influenced preference and satisfaction results (SL-APO dose optimization could be continued at home without direct observation, whereas SC-APO dose optimization took place in clinic), the totality of results from this study and others support patient preference for sublingual administration of apomorphine and may be useful for clinicians when selecting an acute, intermittent treatment for OFF episodes.

The safety profiles of SL-APO and SC-APO were generally comparable and well tolerated. During dose optimization, rates of overall AEs, serious AEs, and AEs leading to discontinuation were comparable between SL-APO and SC-APO. The most common AEs were also generally similar. However, higher rates of nausea were observed during dose optimization with SL-APO (31.4%) compared with SC-APO (22.7%). Despite higher rates of nausea for patients treated with SL-APO, the majority of events were mild or moderate in severity and did not lead to discontinuation of treatment, and none were considered serious. Rates of vomiting during dose optimization were relatively low. Daily solicitation of tolerability during SL-APO dose optimization may have contributed to a bias towards higher rates of nausea in patients receiving SL-APO during that study phase, as rates of nausea were comparable during the treatment phase (SL-APO, 14.1%; SC-APO, 15.7%). Although domperidone use could have mitigated nausea and vomiting, its use was not standardized across the study; however, the proportions of patients who did not utilize antiemetics were similar during treatment with SC-APO and SL-APO in both phases of the study. Further, it is not possible to draw conclusions about prophylactic versus reactive antiemetic use as the database did not capture the time or reason an antiemetic was taken. A previously published study demonstrated that among 43.7% of patients who did not use an antiemetic during SL-APO dose optimization, 86.2% achieved an effective and tolerable dose [31]. Likewise, an interim analysis of the recently completed, open-label, long-term, phase 3 study of SL-APO (*N* = 425) demonstrated that nausea and vomiting rates were similar in patients who did versus did not use antiemetics [32]; additional information about antiemetic use and nausea and vomiting may be analyzed in the future.

Expected differences in route of administration AEs were also noted. Among patients treated with SC-APO, 6.2% of patients reported injection site erythema and 2.1% reported injection site hematoma during dose optimization, and 7.1% and 27.1%, respectively, reported these events during the treatment phase. In patients treated with SL-APO, no individual events in the category of oral application-related AEs occurred at a rate >3% during either study phase; the most frequent oral application-related AEs were lip swelling, mouth ulceration, and stomatitis. The combined incidence of all oral AE terms was 1.0% during dose optimization and 9.9% during the treatment phase. The shorter, 4-week duration of exposure in this study may have influenced the occurrence of oral AEs as compared with a previously published SL-APO study that involved an open-label titration phase followed by a 12-week double-blind maintenance phase [20]. An open-label, long-term study of SL-APO (NCT02542696) was recently completed, and results may provide additional information on the occurrence of oral AEs during long-term SL-APO treatment.

There are several limitations to consider. The primary and secondary endpoints were assessed using a statistical hierarchy. As the primary endpoint was not statistically significant, all subsequent endpoints were evaluated descriptively. Efficacy endpoints were assessed by a rater blinded to treatment assignment, and every effort was made to maintain that blind by preventing the rater from witnessing dosing, covering visible evidence of route of administration, and protecting source data and electronic clinical report forms. However, the open-label administration of SL-APO and SC-APO may have introduced bias. As a direct head-to-head study, there was no placebo group and all patients were on active treatment. As previously discussed, there was no specific data collection method for the use of antiemetics; therefore, the impact of antiemetic use on nausea and vomiting rates is confounded. As SL-APO dose optimization was performed at home by patients, investigators may have been more inclined to increase the dose, whereas this bias may not have been evident for SC-APO dosing performed in clinic under supervision. For a dose to be considered optimal, FULL ON had to occur within 30 min, which may have impacted the optimal dose. Furthermore, doses to which patients were optimized may reflect the investigator and patient’s levels of experience with the treatments, as some patients had experience with SC-APO before study enrollment. Similar to previous studies, patients in the current study administered apomorphine approximately 1.6 times per day, despite reporting four OFF episodes per day, demonstrating that patients do not administer treatments at the same frequency at which they experience OFF episodes [20–22]. This suggests there may be variability in the disability that warrants intervention and/or that patients may have waited for their next dose of maintenance medication rather than treating the OFF episode acutely.

In conclusion, SL-APO demonstrated comparable efficacy for the treatment of OFF episodes associated with PD and a similar safety profile to SC-APO, with patients reporting an overall preference for and greater satisfaction with SL-APO.

## Supplementary Material

Supplementary Material

## Data Availability

Access to de-identified participant data will be provided after a research proposal is submitted online (https://vivli.org) and receives approval from the independent review panel and after a data sharing agreement is in place. Access will be provided for an initial period of 12 months after approval of the data sharing request, but an extension can be granted, when justified, for up to an additional 12 months.

## References

[1] Olanow CW , Stern MB , Sethi K (2009) The scientific and clinical basis for the treatment of Parkinson disease (2009). Neurology 72, S1–S136. 10.1212/WNL.0b013e3181a1d44c 19470958

[2] Carbone F , Djamshidian A , Seppi K , Poewe W (2019) Apomorphine for Parkinson’s disease: Efficacy and safety of current and new formulations. . CNS Drugs 33, 905–918. 31473980 10.1007/s40263-019-00661-z PMC6776563

[3] Chou KL , Stacy M , Simuni T , Miyasaki J , Oertel WH , Sethi K , Fernandez HH , Stocchi F (2018) The spectrum of “off” in Parkinson’s disease: What have we learned over 40 years? Parkinsonism Relat Disord 51, 9–16. 29456046 10.1016/j.parkreldis.2018.02.001

[4] Armstrong MJ , Okun MS (2020) Diagnosis and treatment of Parkinson disease: A review. . JAMA 323, 548–560. 32044947 10.1001/jama.2019.22360

[5] Fox SH , Katzenschlager R , Lim SY , Barton B , de Bie RMA , Seppi K , Coelho M , Sampaio C (2018) International Parkinson and movement disorder society evidence-based medicine review: Update on treatments for the motor symptoms of Parkinson’s disease. . Mov Disord 33, 1248–1266. 29570866 10.1002/mds.27372

[6] Olanow CW , Poewe W , Rascol O , Stocchi F (2021) On-demand therapy for OFF episodes in Parkinson’s disease. . Mov Disord 36, 2244–2253. 34363424 10.1002/mds.28726

[7] Stocchi F (2003) Prevention and treatment of motor fluctuations. . Parkinsonism Relat Disord 9, 73–81. 10.1016/s1353-8020(03)00021-x 12915071

[8] Stocchi F , Tagliati M , Olanow CW (2008) Treatment of levodopa-induced motor complications. , . Mov Disord 23, S599–S612. 18781681 10.1002/mds.22052

[9] Elmer LW , Juncos JL , Singer C , Truong DD , Criswell SR , Parashos S , Felt L , Johnson R , Patni R (2018) Pooled analyses of phase III studies of ADS-5102 (amantadine) extended-release capsules for dyskinesia in Parkinson’s disease. . CNS Drugs 32, 387–398. 29532440 10.1007/s40263-018-0498-4 PMC5934466

[10] Ferreira JJ , Lees A , Rocha J-F , Poewe W , Rascol O , Soares-da-Silva P (2016) Opicapone as an adjunct to levodopa in patients with Parkinson’s disease and end-of-dose motor fluctuations: A randomised, double-blind, controlled trial. . Lancet Neurol 15, 154–165. 26725544 10.1016/S1474-4422(15)00336-1

[11] LeWitt PA , Guttman M , Tetrud JW , Tuite PJ , Mori A , Chaikin P , Sussman NM (2008) Adenosine A2A receptor antagonist istradefylline (KW-6002) reduces “off” time in Parkinson’s disease: A double-blind, randomized, multicenter clinical trial (6002-US-005). . Ann Neurol 63, 295–302. 18306243 10.1002/ana.21315

[12] Lieberman A , Ranhosky A , Korts D (1997) Clinical evaluation of pramipexole in advanced Parkinson’s disease: Results of a double-blind, placebo-controlled, parallel-group study. . Neurology 49, 162–168. 9222185 10.1212/wnl.49.1.162

[13] Pahwa R , Stacy MA , Factor SA , Lyons KE , Stocchi F , Hersh BP , Elmer LW , Truong DD , Earl NL (2007) Ropinirole 24-hour prolonged release: Randomized, controlled study in advanced Parkinson disease. . Neurology 68, 1108–1115. 17404192 10.1212/01.wnl.0000258660.74391.c1

[14] Rajput AH , Martin W , Saint-Hilaire MH , Dorflinger E , Pedder S (1997) Tolcapone improves motor function in parkinsonian patients with the “wearing-off” phenomenon: A double-blind, placebo-controlled, multicenter trial. . Neurology 49, 1066–1071. 9339691 10.1212/wnl.49.4.1066

[15] Rascol O , Brooks DJ , Melamed E , Oertel W , Poewe W , Stocchi F , Tolosa E (2005) Rasagiline as an adjunct to levodopa in patients with Parkinson’s disease and motor fluctuations (LARGO, Lasting effect in Adjunct therapy with Rasagiline Given Once daily, study): A randomised, double-blind, parallel-group trial. . Lancet 365, 947–954. 15766996 10.1016/S0140-6736(05)71083-7

[16] Rinne UK , Larsen JP , Siden A , Worm-Petersen J (1998) Entacapone enhances the response to levodopa in parkinsonian patients with motor fluctuations. Nomecomt Study Group. . Neurology 51, 1309–1314. 9818851 10.1212/wnl.51.5.1309

[17] Thach A , Zichlin M , Peddle M , Du M , Lerner A , Kirson N , Bowling A , Mehta D , Williams R (2002) Systematic literature review of key outcomes used to assess adjunctive treatments for Parkinson’s disease [abstract].. Mov Disord (37 Suppl 1), 783.

[18] Boyle A , Ondo W (2015) Role of apomorphine in the treatment of Parkinson’s disease. . CNS Drugs 29, 83–89. 25676564 10.1007/s40263-014-0221-z

[19] Unti E , Ceravolo R , Bonuccelli U (2015) Apomorphine hydrochloride for the treatment of Parkinson’s disease. . Expert Rev Neurother 15, 723–732. 26037961 10.1586/14737175.2015.1051468

[20] Olanow CW , Factor SA , Espay AJ , Hauser RA , Shill HA , Isaacson S , Pahwa R , Leinonen M , Bhargava P , Sciarappa K , Navia B , Blum D (2020) Apomorphine sublingual film for off episodes in Parkinson’s disease: A randomised, double-blind, placebo-controlled phase 3 study. . Lancet Neurol 19, 135–144. 31818699 10.1016/S1474-4422(19)30396-5

[21] Dewey RB Jr. , Hutton JT , LeWitt PA , Factor SA (2001) A randomized, double-blind, placebo-controlled trial of subcutaneously injected apomorphine for parkinsonian off-state events. . Arch Neurol 58, 1385–1392. 11559309 10.1001/archneur.58.9.1385

[22] Agbo F , Isaacson SH , Gil R , Chiu YY , Brantley SJ , Bhargava P , Navia B (2021) Pharmacokinetics and comparative bioavailability of apomorphine sublingual film and subcutaneous apomorphine formulations in patients with Parkinson’s disease and “OFF” episodes: Results of a randomized, three-way crossover, open-label study. . Neurol Ther 10, 693–709. 33991326 10.1007/s40120-021-00251-6 PMC8571442

[23] Ervin C , Thach A , Lee A , Navia B , Evans E , Doward L (2019) PND100 refinement of the treatment preference questionnaire in adults with Parkinson’s disease and OFF-episodes. Value Health 22, S756.

[24] Atkinson MJ , Sinha A , Hass SL , Colman SS , Kumar RN , Brod M , Rowland CR (2004) Validation of a general measure of treatment satisfaction, the Treatment Satisfaction Questionnaire for Medication (TSQM), using a national panel study of chronic disease. . Health Qual Life Outcomes 2, 12. 14987333 10.1186/1477-7525-2-12 PMC398419

[25] Horváth K , Aschermann Z , Acs P , Deli G , Janszky J , Komoly S , Balazs E , Takacs K , Karadi K , Kovacs N (2015) Minimal clinically important difference on the Motor Examination part of MDS-UPDRS. . Parkinsonism Relat Disord 21, 1421–1426. 26578041 10.1016/j.parkreldis.2015.10.006

[26] Hui JS , Fox SH , Neeson W , Bhargava P , Pappert E , Blum D , Navia B (2020) Open-label titration of apomorphine sublingual film in patients with Parkinson’s disease and “OFF” episodes. . Parkinson Relat Disord 79, 110–116. 10.1016/j.parkreldis.2020.08.028 32927285

[27] Isaacson SH , Bowling A , Zhang I , Pappert E , Stocchi F , CTH-300 and CTH-301 Study Investigators (2023) Motor response with apomorphine sublingual film and levodopa in patients with OFF episodes. . Neurodegener Dis Manag 13, 75–84. 36562349 10.2217/nmt-2022-0038

[28] Pfeiffer RF , Gutmann L , Hull KL Jr. , Bottini PB , Sherry JH , APO302 Study Investigators (2007) Continued efficacy and safety of subcutaneous apomorphine in patients with advanced Parkinson’s disease. . Parkinsonism Relat Disord 13, 93–100. 17055329 10.1016/j.parkreldis.2006.06.012

[29] Thach A , Sutphin J , Coulter J , Leach C , Pappert E , Mansfield C (2021) Patient preferences for treating “OFF” episodes in Parkinson’s disease: A discrete choice experiment. . Patient Prefer Adherence 15, 1187–1196. 34103902 10.2147/PPA.S301644 PMC8179791

[30] Lenton N , Singh S , Szafranski K , Situ A , Thach A , Pappert E , Williams GR (2020) PND2 indirect treatment comparisons of apomorphine sublingual film versus apomorphine subcutaneous injection for the on-demand treatment of “OFF” episodes in patients with Parkinson’s disease. Value Health 23, S623.

[31] Hauser RA , Ondo WG , Zhang Y , Bowling A , Navia B , Pappert E , Isaacson SH (2023) Dose optimization of apomorphine sublingual film for OFF episodes in Parkinson’s disease: Is the prophylactic use of an antiemetic necessary” . J Parkinsons Dis 13, 403–414. 36970914 10.3233/JPD-223537 PMC10200146

[32] Ellenbogen A , Nicholas A , Hauser R , Bhargava P , Pappert E , Navia B (2020) Apomorphine sublingual film for on-demand treatment of “OFF” episodes in patients with Parkinson’s disease: Impact of concomitant antiemetics and dopamine agonists on nausea and vomiting [abstract].. Mov Disord. (35 Suppl 1), S393.

