# Supplementary material for: Apomorphine Sublingual Film Compared with Subcutaneous Apomorphine for OFF Episodes in Parkinson’s Disease: An Open-Label, Randomized, Crossover Study
Source: J Parkinsons Dis. 2023 Dec 19;13(8):1329–42. doi: 10.3233/JPD-230072 (PMC10741320; doi:10.3233/JPD-230072)
Supplement: Supplementary Material [file jpd-13-jpd230072-s001.pdf]

Supplementary Material

Apomorphine Sublingual Film Compared with Subcutaneous Apomorphine for OFF Episodes in Parkinson's Disease: An Open-Label, Randomized, Crossover Study

Supplementary Methods Section

METHODS

Study design and participants

This multicenter, open-label, randomized, crossover study assessed apomorphine sublingual film (SL-APO) compared with subcutaneous apomorphine (SC-APO) in patients with Parkinson's disease (PD) and OFF episodes and was conducted in Europe (EudraCT: 2016-003456-7). Eligible patients were ≥18 years of age with idiopathic PD responsive to and being treated with stable doses of carbidopa/levodopa and any additional PD medications for ≥4 weeks (>8 weeks for monoamine oxidase-B inhibitors), were stage 1–3 by modified Hoehn and Yahr scale when ON, had ≥1 OFF episode/day and ≥2 hours of total daily OFF time, and had a Mini-Mental State Examination score >25. Key exclusion criteria included atypical or secondary parkinsonism; major psychiatric disorder; mouth cankers/sores; prior device-aided treatments; permanent discontinuation of prior SC-APO administration or prior exposure to SL-APO; currently taking selective 5-HT_3 antagonists, selective dopamine antagonists (excluding quetiapine or clozapine), or dopamine-depleting agents; and history of clinically significant impulse control disorders, symptomatic orthostatic hypotension requiring medication, or severe dyskinesia based on Movement Disorder Society Unified Parkinson's Disease Rating Scale (MDS-UPDRS) Part IV.

The study was designed, conducted, and monitored in accordance with the World Medical Association Declaration of Helsinki (1989) and International Council for Harmonisation guidelines. The study protocol and study procedures were approved by institutional review boards and independent ethics committees at each study site: Ethikkommission der Medizinischen Universität Innsbruck, CPP Sud-Ouest et Outre-Mer, Ethikkommission der Universität Ulm, CEIm Fundació de Gestió Sanitària Hospital de la Santa Creu i Sant Pau Servei de Farmacología Clínica, and London-Dulwich Research Ethics Committee. The following local institutional review boards and independent ethics committees were used by investigators, where required: Comitato Etico Campania Sud, Comitato Etico Regionale c/o IRCCS Azienda

Ospedaliera Universitaria San Martino, Comitato Etico Indipendente c/o IRCCS Istituto Clinico Humanitas, Comitato Etico per la Sperimentazione Clinica, Comitato Etico Catania 1 Azienda Ospedaliero-Universitaria, Comitato Etico IRCCS San Raffaele Pisana, Comitato Etico Lazio 2 c/o Segreteria Tecnico Scientifica, Comitato Etico Azienda Ospedaliera Universitaria Università degli Studi della Campania “Luigi Vanvitelli,” Comitato Etico Regionale delle Marche Via Conca, and Comitato Etico Regione Toscana – Area Vasta Nord Ovest. All participants provided written informed consent before any procedures were performed.

Procedures

The study consisted of open-label dose-optimization and open-label treatment phases (Fig. 1). During dose optimization, doses of both medications were optimized in a randomly assigned order to determine the dose that provided a FULL ON (benefit with regard to mobility, stiffness, and slowness and the patient having adequate motor function to perform normal daily activities) within 30 minutes when patients were in a practically defined OFF (no antiparkinson medications after midnight the night before). A washout of 3–7 days occurred between treatment periods.

SL-APO (Supplementary Figure 1) was initiated at 10 mg in clinic, with monitoring of vital signs including blood pressure and pulse, assessed predose and within 60 minutes postdose. If a FULL ON was not achieved within 30 minutes, up-titration (5-mg dose increases; 30-mg dose maximum) during subsequent practically defined OFF episodes could continue at home without direct in-person observation or vital sign monitoring. Clinic staff contacted patients daily by phone during home dose optimization to monitor progress and assess tolerability based on patient self-report. An in-clinic dose-confirmation visit took place after the patient identified their optimal dose at home to confirm the effectiveness and tolerability of the selected dose. Assessments occurring at dose-confirmation visits were the same as those that occurred at the initial in-clinic dose-optimization phase visit. If the investigator determined the FULL ON response to be inadequate (based on effectiveness) or there were tolerability concerns, dose adjustment could continue either in clinic or at home, followed by additional dose-confirmation visits, as needed.

Dose optimization of SC-APO (Supplementary Figure 2) was initiated at 2 mg, the lowest dose, for patients with no previous SC-APO experience and took place entirely in clinic under

direct supervision, with monitoring of vital signs predose and within 60 minutes postdose. Patients with previous SC-APO experience completed a washout of ≥1 day before study enrollment and began dose optimization at the same dose of SC-APO they were taking before screening. If a FULL ON was not achieved within 30 minutes, up-titration in 1-mg increments continued in clinic during subsequent OFF episodes, no earlier than 60 minutes after the prior dose. If a FULL ON was not achieved at the 4-mg dose, the patient returned to the clinic the next day. Dose optimization continued in the same manner until a FULL ON was achieved (maximum 6 mg).

Initially, use of the antiemetic domperidone was optional if clinically warranted and was not to be used prophylactically. After a protocol amendment, domperidone use remained optional but could also be used prophylactically or if clinically warranted at the discretion of the investigator. If initiated, antiemetic therapy was discontinued when judged clinically appropriate.

After a 3- to 7-day washout, patients entered the treatment phase and were randomized in a 1:1 ratio to 4 weeks of treatment with the optimized dose of SL-APO or SC-APO, followed by a washout and an additional 4 weeks of crossover treatment. Patients continued their regular PD medication regimen and could self-administer study treatment for ≤5 OFF episodes per day when needed. Daily dosing of the study drug was recorded in patient diaries. During the 4-week treatment period, patients returned to the clinic for safety and efficacy assessments at 2-week intervals. Patients attended clinic visits in the morning in a practically defined OFF. During the 4-week treatment period, patients returned to the clinic for safety and efficacy assessments at 2-week intervals. Patients attended clinic visits in the morning in a practically defined OFF episode.

Evaluations

The primary efficacy endpoint was change from predose to 90 minutes postdose in MDS-UPDRS Part III score after 4 weeks of dosing in each crossover period, assessed in clinic by a rater blinded to treatment assignment. The blind was maintained by ensuring that the rater did not witness in-clinic dosing, that visible injection sites were covered, and that source data and electronic clinical report forms were protected. Because SL-APO can leave a blue residue on the tongue, a sublingual placebo was administered upon SC-APO in-clinic dosing. Secondary endpoints were evaluated in a hierarchical order and included the following: investigator-rated

durability of response (defined as investigator-confirmed achievement of a FULL ON within 30 minutes postdose and maintenance of that response at 90 minutes postdose) after 4 weeks of dosing in each crossover period, assessed in clinic by a rater blinded to treatment assignment; treatment preference for SL-APO, measured with a patient self-reported Treatment Preference Questionnaire (TPQ; 9-item questionnaire developed for this study to explore patients' experience with and preference for SL-APO compared with SC-APO [1]; Supplementary Table 1) administered after both regimens had been completed; patient-rated durability of response (defined as patient-confirmed achievement of a FULL ON within 30 minutes postdose and maintenance of that response at 90 minutes postdose); and Patient Global Impression of Change to assess patient-rated improvement of OFF, administered after 4 weeks of dosing in each crossover period. Other endpoints included change in MDS-UPDRS Part III score over time (15–120 minutes); investigator-rated time to FULL ON and time to partial ON (period of time where medication is providing some improvement with regard to mobility, stiffness, and slowness but the patient does not have adequate motor function to perform normal daily activities); and patients' general level of satisfaction with medication after 4 weeks of dosing in each crossover period using the validated 14-item Treatment Satisfaction Questionnaire for Medication using a 7-point Likert scale (higher values indicated greater satisfaction) [2].

Pharmacokinetic (PK) concentration-time data for apomorphine and metabolites (apomorphine sulfate, norapomorphine, and others as deemed necessary) was evaluated and PK parameters (including C_{max}, t_{max}, AUC, parent-to-metabolite ratios of C_{max} and AUC) were estimated by noncompartmental methods from plasma samples using actual elapsed time from dosing. The collection of PK samples took place just before dosing and at 15, 30, 60, 90, 120, 180, and 240 minutes postdose (±5 minutes) during the treatment phase.

Unblinded safety evaluations conducted during both study phases included assessments of adverse events, physical examinations, 12-lead electrocardiograms, and vital signs.

Statistical analysis

The primary objective of the study was to demonstrate superiority of SL-APO over SC-APO in improving motor function assessed as change from predose to 90 minutes postdose in MDS-UPDRS Part III score for SL-APO compared with SC-APO after 4 weeks of dosing in each crossover period (primary endpoint). The sample size calculation was based on randomization of

106 patients in the dose-optimization phase and ≥80 patients in the treatment phase, with ≥55 patients expected to complete treatment; this would provide 90% power to detect a mean treatment difference between SL-APO and SC-APO of 5.5 points for the change in MDS-UPDRS Part III score, assuming a standard deviation of 12 points for the period differences in treatment. The primary endpoint was analyzed in the treatment phase modified intention-to-treat population (all patients who were randomized and received ≥1 dose of either study drug in the treatment phase) and was compared between treatment groups using a linear mixed model, with treatment group, visit week (0, 2, 4), treatment by visit week interaction, treatment phase sequence, and period as fixed factors and the week 0 visit predose MDS-UPDRS Part III score as a covariate. The primary and secondary endpoints were tested in a hierarchical order to maintain an overall type I error rate of 0.05. Predicted response rate estimates for durability of effect were based on a generalized linear random effects model and analyzed by the Kenward-Rogers method. Overall treatment preference per the TPQ was originally evaluated on a visual analog scale (VAS) and, following a protocol amendment, was subsequently evaluated on a Likert scale and a VAS. Results from the Likert scale were dichotomized to "preference" or "not preference" and combined with data from the VAS. A missing data imputation rule was applied for patients who discontinued from the study as follows: if a patient terminated the study early, then the early termination (ET) visit was used for analysis; if the ET assessment was not available, then the last treatment received before termination was considered the treatment that was not preferred. The *P* value from a 1-sample, 2-sided test of the null hypothesis that the true proportion is 50% was calculated to evaluate whether a significantly higher proportion of patients preferred SL-APO or not. The confidence interval (CI) and *P* values were calculated using binomial distribution with normal approximation (Wald asymptotic CI). Adverse events were summarized descriptively for both the dose-optimization and treatment phase safety populations (all patients who received ≥1 dose of either study drug for each phase).

Data availability

Access to de-identified participant data will be provided after a research proposal is submitted online (https://vivli.org) and receives approval from the independent review panel and after a data sharing agreement is in place. Access will be provided for an initial period of 12

months after approval of the data sharing request, but an extension can be granted, when justified, for up to an additional 12 months.

REFERENCES

[1] Ervin C, Thach A, Lee A, Navia B, Evans E, Doward L (2019) PND100 refinement of the treatment preference questionnaire in adults with Parkinson's disease and OFF-episodes. *Value Health* **22**, S756.

[2] Atkinson MJ, Sinha A, Hass SL, Colman SS, Kumar RN, Brod M, Rowland CR (2004) Validation of a general measure of treatment satisfaction, the Treatment Satisfaction Questionnaire for Medication (TSQM), using a national panel study of chronic disease. *Health Qual Life Outcomes* **2**, 12.

Supplementary Figure 1. Dose-optimization schematic for apomorphine sublingual film.

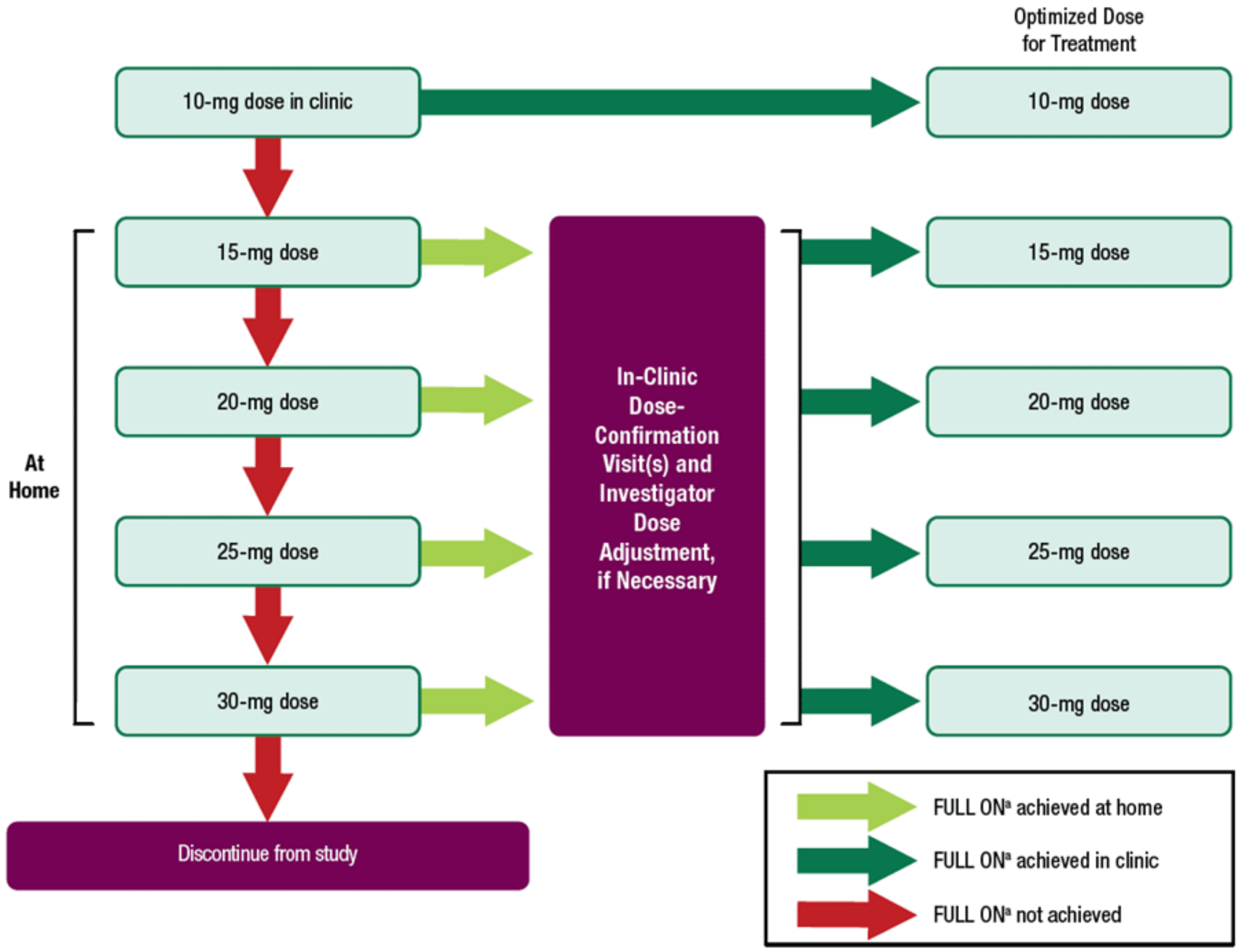

[a]FULL ON was defined as the period when medication provided benefit with regard to mobility, stiffness, and slowness and the patient having adequate motor function to perform normal daily activities.

Supplementary Figure 2. Dose-optimization schematic for subcutaneous apomorphine.

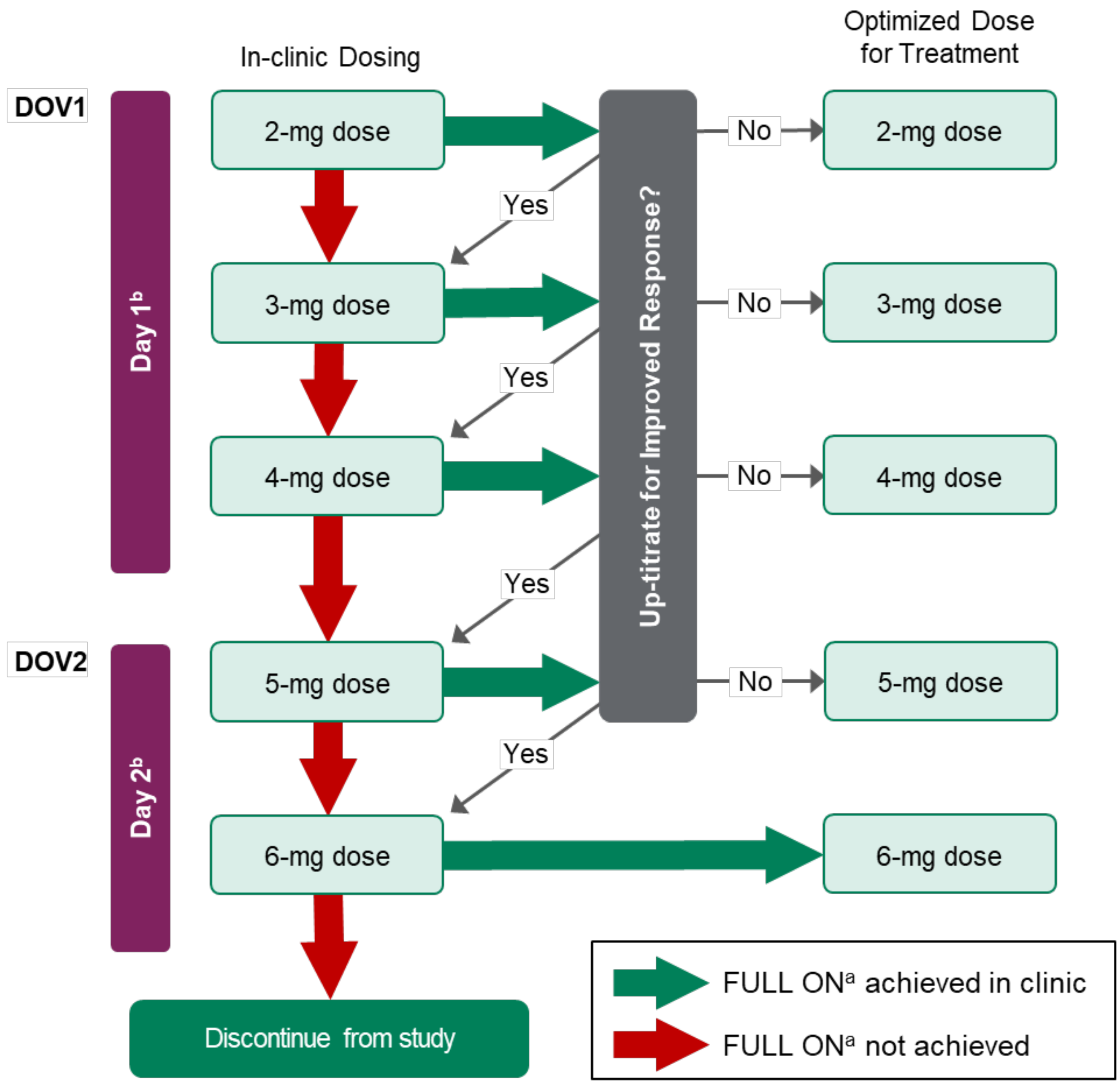

[a]FULL ON was defined as the period when medication provided benefit with regard to mobility, stiffness, and slowness and the patient having adequate motor function to perform normal daily activities.

[b]Patients could receive up to 3 doses per day during subsequent OFF episodes, no sooner than 1 hour after the previous dose.

DOV, dose-optimization visit.

Supplementary Figure 3. Investigator-rated FULL ON[a] (A) and partial ON[b] (B) at week 4 over time (treatment phase mITT population).

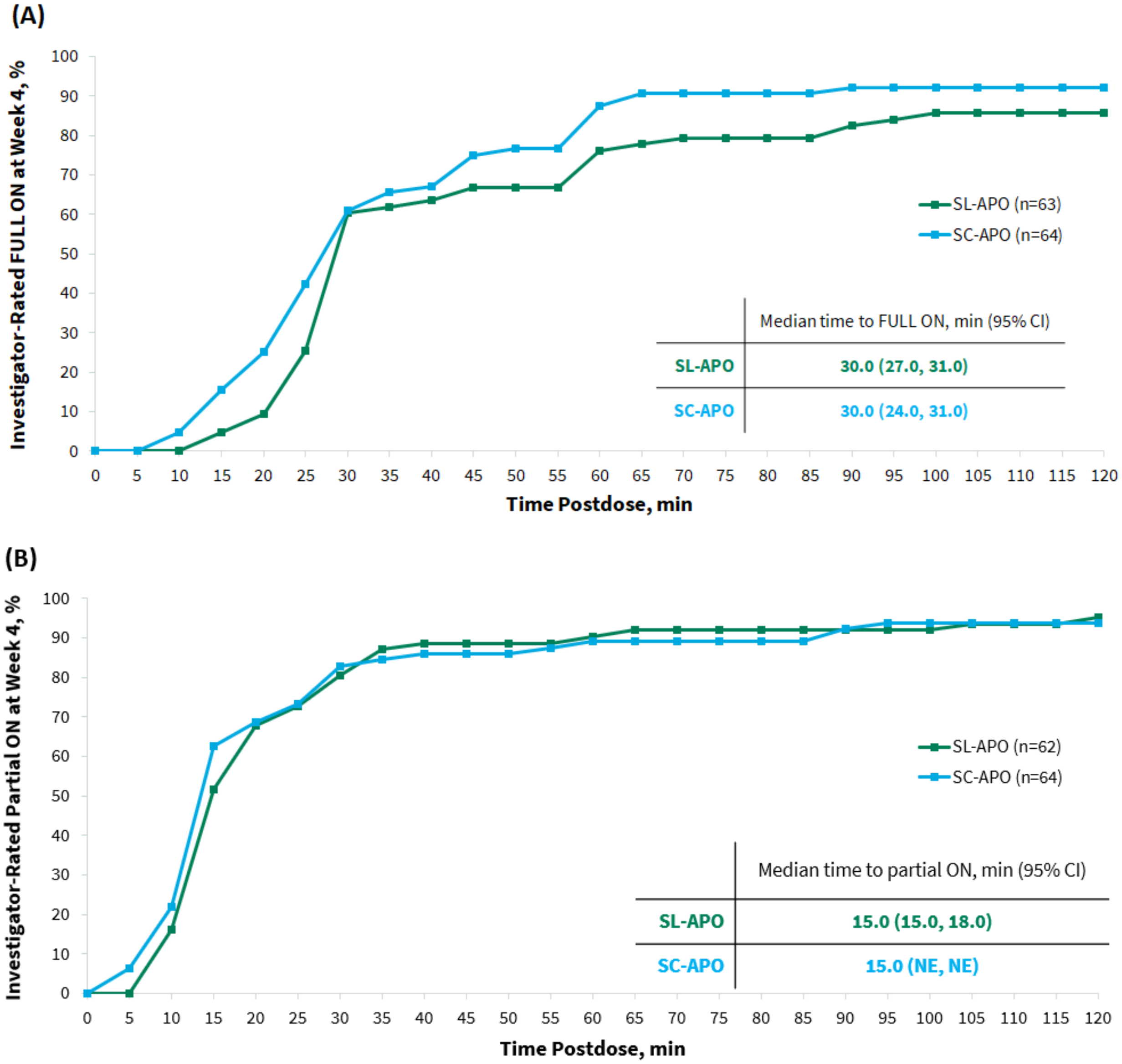

[a]FULL ON was defined as the period when medication provided benefit with regard to mobility, stiffness, and slowness and the patient having adequate motor function to perform normal daily activities.

[b]Partial ON was defined as the period when medication provided some level of improvement with regard to mobility, stiffness, and slowness, but the patient did not have adequate motor function to perform normal daily activities.

CI, confidence interval; mITT, modified intention-to-treat; NE, not evaluable; SC-APO, subcutaneous apomorphine; SL-APO, apomorphine sublingual film.

Supplementary Table 1. Treatment preference questionnaire

Assessment	Question topic
Convenience	• Ease of using the treatment (Q1)<br>• Ability to use the treatment anytime, anywhere (Q3)
Side effects	• Level of troublesome side effects (Q2)
Improvement in motor symptoms	• How quickly the treatment worked on my OFF symptoms (Q4)<br>• How long the effects of treatment lasted on my OFF symptoms (Q5)<br>• How long the treatment allowed me to do my usual daily activities (Q6)<br>• The overall effect of the treatment on my OFF symptoms (Q8)
Overall satisfaction	• My overall satisfaction with the treatment (Q7)
Summary	• Overall, the treatment I prefer for my OFF symptoms is: (Q9)

Q, question.

Supplementary Table 2. CTH-302 Study investigators

Investigator name	Institution	Province	Country
Johannes Schwarz	Kliniken Kreis Mühldorf a. Inn Klinik Haag i. OB	Haag	Germany
Ernest Balaguer Martinez	Hospital Universitari General de Catalunya	Sant Cugat del Vallès	Spain
Jan Kassubek	Universitätsklinikum Ulm, Neurologische Universitätsklinik	Ulm	Germany
Fabrizio Stocchi	IRCCS San Raffaele Pisana - Clinical Trial Center	Rome	Italy
Bettina Wieder	Curiositas ad Sanum Studien und Beratungs GmbH	Munich	Germany
Maria Francesca De Pandis	Centro Ricerche San Raffaele	Cassino	Italy
Lydia Lopez Manzanares	Hospital Universtario de La Princesa, Servicio de Neurologia	Madrid	Spain
Werner Poewe	Medical University Innsbruck - Neurology Department	Innsbruck	Austria
Valentina Leta	Kings College, The Maurice Wohl Neuroscience Institute	London	United Kingdom
Jason Raw	Fairfield General Hospital	Bury	United Kingdom
Alessandro Tessitore	AOU University of Campania "Luigi Vanvitelli", Department of Advanced Medical and Surgical Sciences	Naples	Italy
Maria Jose Marti	Hospital Clinic de Barcelona	Barcelona	Spain
Michele Matarazzo	CINAC, Hospital Universitario HM Puerta del Sur Av. Carlos V, 72, Semisótano, Área de Ginecología Consulta de Enfermería de EECC	Madrid	Spain
Esther Cubo Delgado	Hospital Universitario de Burgos	Burgos	Spain
Maria Gabriela Ceravolo	Ospedali Riuniti di Ancona - Presidio Umberto I; Clinica di Neuroriabilitazione	Ancona	Italy
Olivier Rascol	Centre d'Investigation Clinique, CIC 1436, CHU Purpan Hôpital Pierre-Paul Riquet	Toulouse	France
Siegfried Muhlack	St. Josef-Hospital, Klinikum der Ruhr-Universität-Bochum, Neurologische Klinik	Bochum	Germany
Sophie Molloy	Imperial College Healthcare Trust NHS Charing Cross Hospital	London	United Kingdom
Walter Pirker	Wilhelminenspital	Vienna	Austria
Giovanni Castelnovo	CHU Carémeau, Service de neurologie	Nimes	Germany
Florin Gandor	Kliniken Beelitz GmbH Neurologisches Fachkrankenhaus für Bewegungsstörungen / Parkinson	Beelitz-Heilstätten	Germany
Andrea Keuhn	Charité Universitätsmedizin Berlin - Charité Campus Mitte, Bewegungsstörungen und Neuromodulation	Berlin	Germany
Björn Falkenburger	Universitätsklinikum Carl Gustav Carus an der TU Dresden, Klinik und Poliklinik für Neurologie	Dresden	Germany
Jaime Kulisevsky Bojarski	Hospital de la Santa Creu i Sant Pau	Barcelona	Spain
Camille Carroll	Plymouth University	Plymouth	United Kingdom
Jonathan Evans	Nottingham University Hospitals NHS Trust Queens Medical Centre Campus	Nottingham	United Kingdom
Roberto Ceravolo	Azienda Ospedaliero Universitaria Pisana Neurology	Pisa	Italy
Alexandre Eusebio	Hôpital de la Timone, Service de Neurologie et Pathologie du mouvement	Marseille	France
David Maltete	Rouen University Hospital 1 rue de Germont, Service de Neurologie	Rouen	France

Investigator name	Institution	Province	Country
Thomas Mueller	St Joseph Krankenhaus Berlin-Weissensee, Abteilung für Neurologie	Berlin	Germany
Bernhard Haslinger	Klinikum rechts der Isar der Technischen Universität München, Klinik und Poliklinik für Neurologie	München	Germany
Barone Paolo	A.O.U San Giovanni di Dio e Ruggi d'Aragona	Salerno	Italy
Alberto Albanese	Istituto Clinico Humanitas<br>Dipartimento di Neurologia	Milan	Italy
Elisabetta Gasparoli	IRCCS San Camillo di Venezia - Dipartimento Malattia di Parkinson	Venezia	Italy
Mario Zappia	University Hospital Policlinico-Vittorio Emanuele Department "G.F. Ingrassia", Section of Neurosciences	Catania	Italy
Juan Carlos Gomez Esteban	Hospital Universitario de Cruces	Barakaldo	Spain
